# Administration of the Antioxidant N-Acetyl-Cysteine in Pregnant Mice Has Long-Term Positive Effects on Metabolic and Behavioral Endpoints of Male and Female Offspring Prenatally Exposed to a High-Fat Diet

**DOI:** 10.3389/fnbeh.2018.00048

**Published:** 2018-03-15

**Authors:** Alessandra Berry, Veronica Bellisario, Pamela Panetta, Carla Raggi, Maria C. Magnifico, Marzia Arese, Francesca Cirulli

**Affiliations:** ^1^Center for Behavioral Sciences and Mental Health, Istituto Superiore di Sanità, Rome, Italy; ^2^Department of Biochemical Sciences, Sapienza University of Rome, Rome, Italy

**Keywords:** N-acetyl-cysteine, high-fat diet, pregnancy, metabolism, oxidative stress, behavior, glutathione, mice

## Abstract

A growing body of evidence suggests the consumption of high-fat diet (HFD) during pregnancy to model maternal obesity and the associated increase in oxidative stress (OS), might act as powerful prenatal stressors, leading to adult stress-related metabolic or behavioral disorders. We hypothesized that administration of antioxidants throughout gestation might counteract the negative effects of prenatal exposure to metabolic challenges (maternal HFD feeding during pregnancy) on the developing fetus. In this study, female C57BL/6J mice were fed HFD for 13 weeks (from 5-weeks of age until delivery) and were exposed to the N-acetyl-cysteine (NAC) antioxidant from 10-weeks of age until right before delivery. Body weight of the offspring was assessed following birth, up to weaning and at adulthood. The metabolic, neuroendocrine and emotional profile of the adult offspring was tested at 3-months of age. Prenatal HFD increased mother’s body weight and offspring’s weight at the time of weaning, when administered in conjunction with NAC. In females, NAC administration reduced high levels of leptin resulting from prenatal HFD. Prenatal NAC administration also resulted in greater glucose tolerance and insulin sensitivity while increasing adiponectin levels, as well as increasing exploratory behavior, an effect accompanied by reduced plasma corticosterone levels in response to restraint stress. Analysis of glutathione levels in the hypothalamus and in brown adipose tissue indicates that, while HFD administration to pregnant dams led to reduced levels of glutathione in the offspring, as in the male hypothalamus, NAC was able to revert this effect and to increase glutathione levels both in the periphery (Brown Adipose Tissue, both males and females) and in the central nervous system (males). Overall, results from this study indicate that the body redox milieu should be tightly regulated during fetal life and that buffering OS during pregnancy can have important long-term consequences on metabolic and behavioral endpoints.

## Introduction

In Western society the consumption of high fat diet (HFD) as well as a sedentary lifestyle has been associated with metabolic derangements and impaired brain plasticity (Schrauwen and Westerterp, 2000; Unger and Orci, 2001; McGarry, 2002; Molteni et al., 2002, 2004). While it is generally accepted that HFD-induced obesity *per se* is associated with adverse health outcomes, some studies have also shown that maternal obesity is specifically associated with a variety of pregnancy complications, fetal and neonatal death, maternal hypertension and gestational diabetes (Lu et al., 2001; Bhattacharya and Sood, 2007; Thompson, 2008). Moreover, it is possible to hypothesize that maternal obesity might increase in the offspring, vulnerability for cardiovascular or metabolic pathological conditions, in addition to the often comorbid mood-disorders. Indeed, evidence suggests that epigenetic events initiated by HFD exposure during the prenatal period can result in persistent adaptations in physiological and metabolic regulations of the developing organism, predisposing the offspring towards disease (Barker, 1995; Lucas, 1998). Furthermore, HFD may negatively impact on metabolic regulation resulting in excess energy intake and increased adiposity (Woods et al., 2004; Zhang et al., 2009) also associated to low-grade inflammation and increased production of inflammatory cytokines (Chandalia and Abate, 2007).

Several studies have shown that administration of high fat or high calorie diets to rodents increases the generation of reactive oxygen species (ROS; Zhang et al., 2005) and protein oxidation (Souza et al., 2007), thus resulting in increased oxidative damage in the brain (Cecarini et al., 2007; Bruce-Keller et al., 2009; Zhang et al., 2009) and, more in general, in oxidative cell injury (Gutteridge and Halliwell, 2000). This evidence is consistent with the hypothesis that increased oxidative stress (OS) may mediate the effects of HFD consumption both on brain health and mood disorders and may as well exacerbate the HFD-related metabolic impairment.

In a previous work performed in mice (Bellisario et al., 2014), we found that the detrimental effect of prenatal exposure to a maternal HFD was counteracted by the lack of the p66Shc gene that results, among other several features, in reduced OS and resistance to diet-induced obesity. More in detail, p66Shc^−/−^ mice showed blunted changes in glycaemia levels in response to glucose or insulin challenges and were protected from the disrupting effect of prenatal HFD on neuroendocrine activation in response to a psychophysical stress (Bellisario et al., 2014). These findings suggested that reduced OS might increase resistance to impaired physiological adaptation driven by maternal HFD in the offspring during adult life. In light of this evidence, this study was aimed at developing a pharmacological model of reduced OS able to cope with metabolic challenges occurring during early life by means of prenatal administration of N-acetyl-cysteine (NAC). This compound is the rate-limiting substrate in the biosynthesis of glutathione, is a powerful antioxidant and a ROS scavenger; its clinical efficacy and safety as a mucolytic drug have been widely established in the last decades, both as a therapy for respiratory diseases and as a remedy towards several acute intoxications including the most common acetaminophen drug overdose (Crystal and Bast, 1991; Samuni et al., 2013). NAC is “a safe and well-tolerated supplementary drug without any considerable side effects commonly used in clinical practice for a variety of different disorders ranging from polycystic ovary syndrome, preterm-birth, recurrent pregnancy loss, chronic bronchitis, ulcerative colitis, liver cancer, muscle performance, asthma, Alzheimer and Parkinson” (see Mokhtari et al., 2017 and references therein for a complete review). Furthermore, there is evidence showing that NAC administration during pregnancy might counteract apoptosis and ROS-related genotoxicity by increasing glutathione levels and decreasing mitochondrial membrane depolarization within the cells (Amin et al., 2008).

Our working hypothesis was that administration of the antioxidant NAC during prenatal life might increase glutathione levels, preventing the negative effects exerted by increased ROS resulting from maternal HFD administration on metabolic and behavioral endpoints in the offspring. In addition, a growing body of evidence suggests a direct link between redox imbalance and emotional disturbance, particularly anxiety (Hovatta et al., 2005 and maternal obesity has been also independently related to increased incidence of mood disorders in the offspring (Bouayed et al., 2009; Contu and Hawkes, 2017; Edlow, 2017). Thus, another hypothesis to be tested was that a metabolic stressor, such as prenatal HFD, might lead to increased anxiety in the offspring and that NAC would prevent this effect.

To test such hypothesis female mice were fed HFD or control diet (CD) for 13 weeks (from 5 weeks of age until right before delivery) and were exposed to NAC for 8 weeks (from 10 weeks of age until right before delivery). The offspring was subsequently phenotyped for metabolic and behavioral responses at adulthood.

## Materials and Methods

### Animals

Experimental subjects were male and female adult offspring (3 months of age) of C57Bl/6J female mice fed HFD or CD—see below.

All mice were housed two/cage in transparent Plexiglas cages provided by Tecniplast, in an air-conditioned room (temperature 21 ± 1°C, relative humidity 60 ± 10%), under a reversed 12/12 h light/dark cycle with lights off from 07:00 a.m. to 07:00 p.m. Fresh tap-water and standard chow (standard diet—SD—energy 3.3 kcal/g, fat 17%, carbohydrate 60% and protein 23% provided by Altromin-R, Rieper, Italy) were continuously available until 5 weeks of age.

### Diet Administration

At 5 weeks of age, all females (*n* = 77) were fed *ad libitum* either with HFD (energy 5.56 kcal/g, fat 58%, carbohydrate 25.5% and protein 16.4%; *n* = 43) or CD (energy 4.07 kcal/g, fat 10.5%, carbohydrate 73.1% and protein 16.4%; *n* = 34) for 13 weeks, i.e., until right before delivery. Females were randomly assigned to HFD or CD groups and subjects were counterbalanced to avoid biases due to body weight. High-fat (D12331) and control (D12328) diets were provided by Research Diets Inc., New Brunswick, NJ, USA.

### NAC Antioxidant Administration

After 5 weeks on the diets, females underwent antioxidant treatment with NAC (or tap water as control), for 5 weeks, until the end of dietary treatment. In order to minimize stress due to excessive handling procedure by the experimenter NAC (Sigma-Aldrich) was daily administered in drinking water (Balansky et al., 1996), to yield an average dose of 1 g/kg body weight.

### Breeding Procedure

At 15 weeks of age, after 10 weeks on the diet and 5 weeks of NAC administration, all females were bred (see Bellisario et al., 2014 for further details on the breeding procedure; Brockman et al., 1998).

**Figure 1:**
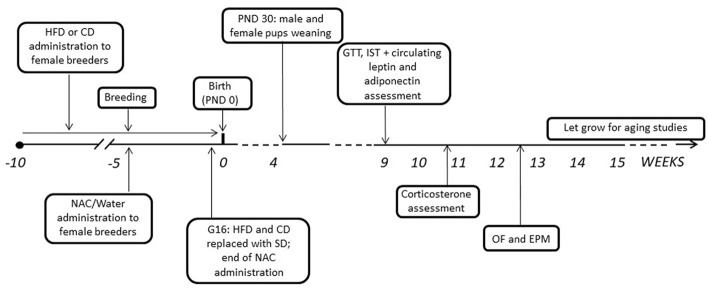
Graphical representation of the experimental timeline. HFD, high-fat diet; CD, control diet; SD, standard diet; NAC, N-acetyl-cysteine; PND, postnatal day; G, gestational day; GTT, Glucose tolerance test; IST, insulin sensitivity test; OF, open field; EPM, elevated plus maze.

After mating, dams were kept with either HFD or CD and NAC throughout gestation until 3 days before the expected delivery date (i.e., gestational day 16—G16); by this time NAC administration was also stopped and all dams were shifted onto SD (see Bellisario et al., 2014). Body weight gain of pregnant females was monitored once a week throughout the 5 weeks of combined exposure to HFD and NAC. Pups’ birth was considered as post-natal day 0 (PND 0). At PND 3 pups’ weight was registered, as well as at PND 30, when all pups were also weaned onto SD. Starting at 9 weeks of age, both male and female offspring were weighed and tested to assess the metabolic and emotional profiles resulting from prenatal exposure to a HFD diet and the role of antioxidants on these regulations. A schematic design of the experimental plan is reported in Figure 1. All experimental procedures were conducted in conformity with the European Directive 2010/63/EU and the Italian legislation on animal experimentation, D.Lgs.vo 26/2014 and were approved by the Istituto Superiore di Sanità Ethical Committee (*Organismo Preposto per il Benessere Animale—OPBA*) and by the Italian Ministry of Health.

## Experimental Procedures

### Metabolic Regulation

#### Glucose Tolerance Test (GTT)

Intra-peritoneal Glucose Tolerance Test (GTT) was performed after a 15 h overnight fasting that took place from 06:30 p.m. to 09:30 a.m. Animals were intra-peritoneally (ip) loaded with 2 g/kg body weight D-glucose (10% D glucose solution; Sigma, St. Louis, MO, USA; Satapathy et al., 2011). Blood was collected from the tail vein at 0 (baseline), 30, 60, 120 and 180 min (Ranieri et al., 2010) following ip injection and glycemia (blood glucose concentration) was measured using a commercial glucometer (StatStrip Xpress-i, nova biomedical, A. Menarini diagnostic; Titta et al., 2010).

#### Insulin Sensitivity Test (IST)

The test was performed on animals starved for 5 h that took place from 09:30 a.m. to 02:30 p.m. Glycemia was measured using a commercial glucometer (StatStrip Xpress-i, nova biomedical, A. Menarini diagnostic) immediately before (0) and 15, 30, 60, 120 min after ip injection of a 0.4 U/kg body weight (Titta et al., 2010) solution of human recombinant insulin (Humulin, Eli-Lilly, 100 U/mL; Ranieri et al., 2010).

#### Metabolic Hormones Assessment

Plasma levels of leptin and adiponectin were assessed under starving condition. Blood samples were collected from the tail vein in potassium EDTA coated tubes (1.6 mgEDTA/mlblood; Sarstedt, Germany) at 09:30 a.m., 15 h after removal of food (see “Glucose Tolerance Test (GTT)” section above). After centrifugation, plasma samples were used for the determination of leptin (MouseLeptin Elisa kit, CrystalChemInc., DownersGrove, IL, USA) and adiponectin levels (Elisa kit, B-BridgeInternational, Inc., Santa Clara, CA, USA).

All subjects underwent the GTT as first metabolic test and after 3 days, in which mice were left undisturbed, all of them were tested for the Insulin Sensitivity Test (IST). This time interval was necessary to recover from the fasting and the handling procedures. Five days following the end of the metabolic measurement subjects underwent behavioral tests.

### Plasma Corticosterone in Response to Restraint Stress

Two weeks following the end of the metabolic assessments, the activation of the hypothalamus-pituitary-adrenal (HPA) axis was assessed in response to a psychophysical stressful challenge. All subjects underwent an acute restraint stress (30 min) and blood samples were collected by a tail nick at different time points, i.e., soon before (0 min) and following (30, 180 and 240 min) the exposure to stress, in order to measure plasma levels of corticosterone (CORT). Exposure to stress took place at 02:30 p.m., when the levels of free CORT were far from the circadian peak (Kitchener et al., 2004). Blood samples (100 ml, approximate volume) were collected individually in potassium EDTA coated tubes (1.6 mg EDTA/ml blood, Sarstedt, Germany). All samples were kept on ice and later centrifuged at 3000 rpm for 15 min at +48°C. Blood plasma was transferred to Eppendorf tubes for CORT determination and stored at −20°C until further analysis. CORT was measured using a commercially available radioimmunoassay (RIA) kit containing ^125^iodine labeled CORT; 5 μl of plasma were sufficient to carry out CORT measurement. Sensitivity of the assay was 0.125 μg/dl, inter- and intra-assay variation was less than 10 and 5%, respectively (MP Biomedicals Inc., CA, USA). Vials were counted for 2 min in a gamma scintillation counter (Packard Minaxi Gamma Counter, Series 5000).

### Measurement of Glutathione in Hypothalamus (HYPO) and Brown Adipose Tissue (BAT)

The amount of total glutathione equivalents (GSH + GSSG) were measured in mouse hypothalamic and brown adipose tissue homogenates by DTNB according to the manufacturer instructions (Glutathione Assay Kit, Cayman Chemicals, Ann Arbor, MI, USA). Briefly, hypothalamic tissues were homogenized in phosphate buffer containing 1 mM EDTA. After centrifugation at 10,000× *g* for 15 min at 4°C, the supernatants were deproteinated by adding an equal volume of metaphosphoric acid. The pH of samples was set in a range of 6–7 by triethanolamine (Sigma-Aldrich). Samples were diluted 1:2 with MES Buffer (0.2 M 2-(N-morpholino) ethanesulfonic acid/0.05 M phosphate/1 mM EDTA) and then assayed for total glutathione quantification by the addition of a mix containing: DTNB, glutathione reductase, NADP^+^, glucose-6-phosphate and glucose-6-phosphate dehydrogenase. The absorbance at 412 nm was detected by the Appliskan Microplate Reader (Thermo Scientific).

### Emotional Phenotype

After 2 weeks of washout from the manipulation required to assess plasma CORT levels, all subjects underwent an open field (OF) followed (2 days later) by an elevated plus maze (EPM) Test in order to assess spontaneous and emotional behavior respectively (Pellow et al., 1985; File, 1993). All tests were performed during the active (dark) phase of the cycle between 09:00 and 13:00.

#### Open Field (OF)

Each subject was individually placed in the center of a cubic arena (OF box 40 × 40 × 40 cm) made of Plexiglas and allowed to freely explore for a single session lasting 15 min. The OF box was ideally divided into 25 squares and ideally partitioned into a central portion (24 × 24 cm) and a peripheral one, identified as the remaining part of the arena. When data were analyzed, the session was subdivided in three time blocks (tb), lasting 5 min, and the time spent in each portion of the arena was measured. Furthermore, the frequency of *sniffing* and *rearing* was scored as index of exploratory behavior and the latency and frequency of *immobility* were considered as index of emotional profile. *Grooming* frequency and the latency to the first event were also taken into account, providing a reliable marker of high or low stress in a specific behavioral context (Katz and Roth, 1979; Kalueff and Tuohimaa, 2005).

#### Elevated Plus Maze (EPM)

The EPM is made of two open arms (30 × 5 × 0 cm) and two closed arms (30 × 5 × 15 cm) that extended from a common central platform (5 × 5 cm). The apparatus, made of Plexiglas (gray floor, clear walls), is elevated to a height of 60 cm above the floor. Mice were individually placed on the central platform facing an open arm and allowed to freely explore the maze for 5 min. Behavioral parameters observed were: % open entries [(open/total) × 100] and time spent in the open and closed arms of the maze (File, 2001). Furthermore, the behavioral parameters taken into account were the frequency of *head-dipping* (HEAD) and of explorative behavior such as *sniffing* and locomotion (*crossings* of squares limits with all paws).

Illumination was provided by means of two tall floor lamps with translucent shades placed at opposite corners of the room providing an equal light intensity between the periphery and the central part of the OF as well as between the open and closed arms. The total luminosity was 400 lux on average. Behavioral performances were video recorded and the behavioral analysis was carried out from the video by an observer blind to the prenatal treatment, using commercial software (The Observer 10XT, Noldus, Netherlands). At the end of each behavioral session apparatuses were cleaned by a cotton pad wetted with a 50% solution of ethanol and water.

### Statistical Analysis

Data were analyzed using parametric analysis of variance (ANOVA) with Treatment (Maternal NAC vs. WATER), Diet (Maternal HFD vs. CD) and Sex (females vs. males) as between-subjects factors and time blocks as within-subjects repeated measures, when appropriate (GTT, IST, CORT and OF tests). “Zones” were also considered as a within-subject factor for the EPM (“center” vs. “closed arms” vs. “open arms”) and the OF (“center” vs. “periphery” as a within-subjects factor) to account for the preference of subjects for the different areas of the maze/arena.

*Post hoc* comparisons were performed using the Tukey’s test. In the case of GTT and IST Tukey’s *post hoc* comparisons were performed also in the absence of significant ANOVA effects (Diet × Treatment × Time course) according to the indications given by Wilcox et al. (1987). When a main effect of sex was found, separate analyses for females and males were performed. A level of probability set at *p* < 0.05 was used as statistically significant. Data are presented graphically as means + SEM. Statistics were performed with Statview II (Abacus Concepts, Berkeley, CA, USA).

## Results

### Dams’ Body Weight

**Figure 2:**
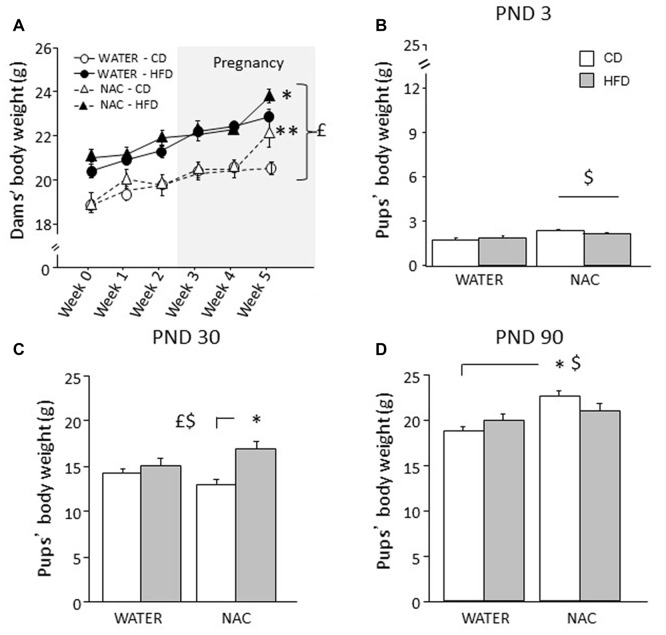
Body weight of dams and offspring. **(A)** HFD increased dams’ body weight during both peri-conceptional time and pregnancy (gray shade) moreover, NAC increased dams’ body weight on week 5. **(B)** Prenatal NAC increased pups’ body weight on PND 3; **(C)** on PND 30 HFD overall increased pups’ body weight particularly when in combination with NAC **(D)** this effect was reverted on PND 90. Data are mean ± SEM (dams) and + SEM (pups); ^$^*p* < 0.05, main effect of NAC; ^£^*p* < 0.05, main effect of HFD; **p < 0.01 NAC-CD vs. NAC-HFD **(A)**; *p < 0.05, NAC-HFD vs. WATER-HFD **(A)**; NAC-HFD vs. NAC-CD **(C)**; NAC-CD vs. WATER-CD **(D)**.

Overall, HFD feeding increased body weight in all dams (main effect of diet: *F*_(1,35)_ = 37.320; *p* < 0.0001). An interaction among treatment, diet and time was found during the last week of combined exposure to HFD and NAC (*F*_(5,175)_ = 2.978; *p* = 0.0132) with NAC-CD and NAC-HFD females showing increased body weight (b.w.) when compared to their respective controls (WATER-CD and WATER-HFD; *post hoc* comparisons *p* < 0.01 and *p* < 0.05 respectively; see Figure 2A).

### Body Weight of the Offspring and Body Mass Index (BMI)

Overall NAC increased the b.w. of the offspring on PND 3 (main effect of NAC: *F*_(1,30)_ = 9.734, *p* = 0.004, Figure 2B) and on PND 90 (*F*_(1,64)_ = 65.011, *p* < 0.0001). By contrast, prenatal exposure to maternal HFD increased offspring’s body weight specifically on PND 30 (main effect of HFD: *F*_(1,63)_ = 13.547, *p* = 0.0005, Figure 2C) but not on PND 3 and PND 90 (main effect of maternal diet: *F*_(1,30)(1,64)_ = 0.104; 0.698, *p* = 0.7489; 0.4065, respectively on PND 3 and on PND 90). In addition, on PND 30 and PND 90 an interaction between maternal diet and NAC was found, with NAC contributing to increase body weight in HFD offspring on PND 30 (*F*_(1,63)_ = 5.828, *p* = 0.0187, Figure 2C), while having an opposite effect on PND 90 (*F*_(1,64)_ = 25.363, *p* < 0.0001, Figure 2D).

As for BMI, while neither NAC or maternal HFD determined differences among groups (main effect respectively of NAC and maternal diet: *F*_(1,55)_ = 2.421; 0.005, *p* = 0.1255; 0.9433), sex strongly affected this parameter with males being characterized by a higher BMI than females (main effect of sex: *F*_(1,55)_ = 19.575, *p* < 0.0001). In addition, an interaction among NAC, HFD and sex (*F*_(1,55)_ = 6.962, *p* = 0.0108) was found with NAC-HFD males showing a higher BMI than females of the same group (data not shown).

### Metabolic Regulation

#### Glucose Tolerance Test (GTT)

**Figure 3:**
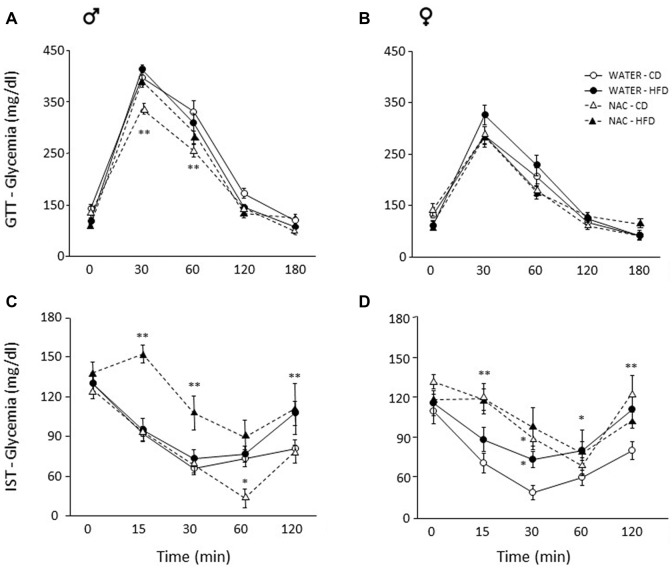
Glucose tolerance test (GTT) and insulin sensitivity test (IST). Glucose tolerance: **(A)** NAC-CD males were characterized by improved glucose tolerance particularly 30 and 60 min following glucose loading. **(B)** No difference was found among treatment groups in females. Insulin sensitivity: **(C)** Interestingly, NAC-CD males showed improved insulin sensitivity 60 min following insulin injection. By contrast, the NAC-HFD subjects showed an overt worsening of the glycaemic profile. **(D)** Females showed a glycaemic profile comparable to that of NAC-HFD comparable males as a result of NAC alone. Data are mean ± SEM; **p < 0.01: **(A)** NAC-CD vs. all other treatment groups at time points 30 and 60. **(C)** NAC-HFD vs. all other treatment groups at time points 15 and 30; WATER-HFD and NAC-HFD vs. WATER-CD and NAC-CD at time point 120; **(D)** NAC-CD vs. WATER-CD at 15 and 120 min. *p < 0.05: **(C)**, NAC-CD vs. all other treatment groups at time point 60; **(D)**, NAC-CD and WATER-HFD vs. WATER-CD at time point 30; WATER-HFD vs. WATER-CD at time point 60.

Overall, a main effect of sex was found (main effect of sex: *F*_(1,64)_ = 92.889, *p* < 0.0001) thus male and female subjects were analyzed independently in order to better characterize the metabolic response in the two sexes. Maternal HFD did not affect glycaemia *per se* in males (main effect of diet: *F*_(1,33)_ = 0.059; *p* = 0.8096) or females (main effect of diet: *F*_(1,31)_ = 0.707, *p* = 0.4069), by contrast, prenatal exposure to NAC improved glucose tolerance in males only (*F*_(1,33)_ = 12.298; *p* = 0.0013). Indeed, although the interaction among Diet, Treatment and Time course failed to show statistical significance (*F*_(4,132)_ = 0.823; *p* = 0.5129), *post hoc* comparisons revealed improved glucose tolerance at 30 and 60 min in NAC-CD subjects (*p* < 0.001; Figure 3A). No interaction effects were observed in females (*F*_(4,124)_ = 1.139; *p* = 0.3414, Figure 3B).

#### Insulin Sensitivity Test (IST)

Since sex interacted significantly with Diet and Treatment (Treatment × Diet × Sex: *F*_(1,62)_ = 8.243, *p* = 0.0056) data were analyzed independently in order to better characterize the metabolic response in the two sexes. In male mice, NAC alone resulted in improved insulin sensitivity, an opposite effect was found when this was administered in combination with HFD (*F*_(1,33)_ = 6.291, *p* = 0.0172, *post hoc* NAC-HFD vs. NAC-CD *p* < 0.01). This insulin resistance effect was observed at each time point following insulin injection (interaction among maternal diet, treatment and time, *F*_(4, 132)_ = 3.087, *p* = 0.0182; Figure 3C).

In females, prenatal NAC overall resulted in decreased insulin sensitivity, regardless of prenatal diet (main effect of NAC: *F*_(1,30)_ = 8.604, *p* = 0.0064, Figure 3D). *Post hoc* comparisons performed on the interaction among treatment, diet and time (*F*_(4,120)_ = 1.598; p = 0.1792) revealed that this effect was apparent at 15 (NAC-CD vs. WATER-CD and NAC-HFD vs. WATER- HFD, *p* < 0.01) and 30 min (NAC-CD vs. WATER-CD, *p* < 0.05) following insulin injection. Moreover, when compared to the control condition (WATER-CD) treatment with HFD led to a worsening of the glycaemic profile at 30 (*p* < 0.05) and at 120 min (*p* < 0.01; WATER- HFD vs. WATER- CD *p* < 0.05). These* post hoc* comparisons were performed in the absence of a significant ANOVA effects (Diet × Treatment × Time course) according to the indications given by Wilcox (Wilcox et al., 1987).

#### Adiponectin Levels

**Figure 4:**
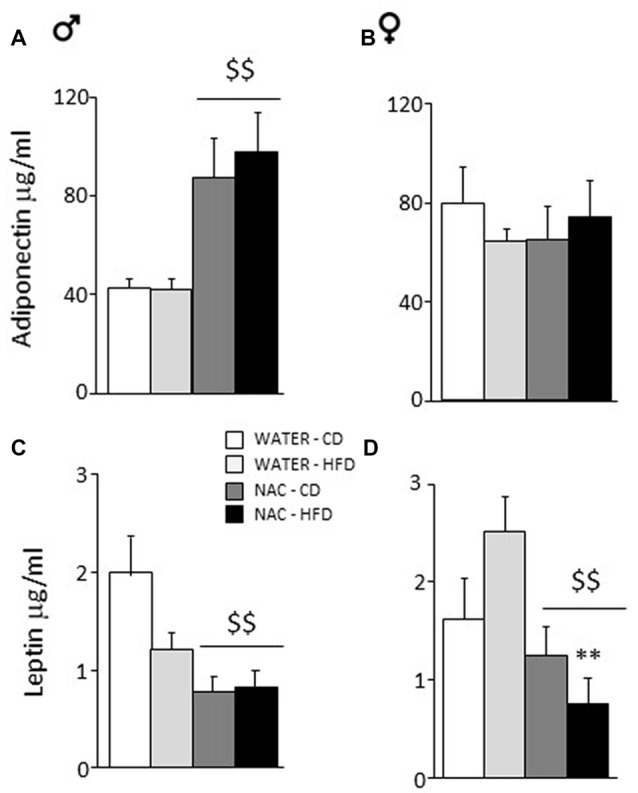
Adiponectin and Leptin plasma levels. **(A)** NAC increased adiponectin in males while **(B)** no effect was observed in females. **(C)** An opposite effect of NAC was observed for leptin levels in males. **(D)** In females, this reduction of leptin levels was much stronger since they were more vulnerable to the effects of HFD. Data are mean + SEM. Main effect of NAC: ^$$^*p* < 0.01 **(A,C,D)**; **p < 0.01 **(D)**, NAC-HFD vs. WATER-HFD (females).

Overall, prenatal treatment with NAC increased circulating adiponectin levels (main effect of treatment: *F*_(1,33)_ = 5.065; *p* = 0.0312) while neither diet or sex affected adiponectin levels *per se* (*F*_(1,33)_ = 0.002; 0.108; *p* = 0.9671; 0.7440, respectively for diet and sex). By contrast, a significant interaction between treatment and sex showed that NAC increased this adipokine in males but not in females (*F*_(1,33)_ = 6.366; *p* = 0.0166; *post hoc*: males-NAC vs. males-WATER *p* < 0.01, Figures 4A,B).

####  Leptin Levels

Overall, prenatal treatment with NAC decreased circulating leptin levels (main effect of treatment: *F*_(1,45) =_ 18.316; *p* < 0.0001). Moreover, NAC not only decreased leptin levels in males but was particularly effective in preventing the HFD-driven increase in leptin levels in female offspring (interaction among treatment, diet and sex: *F*_(1,45)_ = 6.393; *p* = 0.0150, *post hoc*: water-HFD-females vs. NAC-HFD-females, *p* < 0.01, see Figures 4C,D).

### Plasma Corticosterone in Response to Restraint Stress

As a main effect of sex was found (*F*_(1,55)_ = 9.335, *p* = 0.0035), a further analysis was ran separately for males and females.

**Figure 5:**
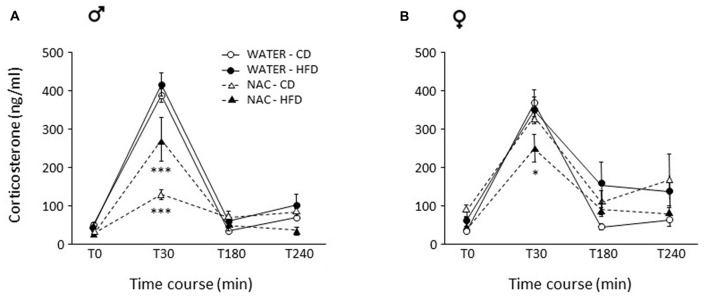
Activation of the hypothalamus-pituitary-adrenal (HPA) axis after restraint stress. **(A)** In males, NAC led to overall decreased corticosterone (CORT) levels. This decrease was stronger in NAC-CD subjects, maternal HFD reducing this effect. **(B)** In females, the combination of prenatal NAC and HFD led to a reduction in CORT levels comparable to that observed in NAC-HFD males while NAC alone did not affect this parameter. Data are mean ± SEM. ***p < 0.001 **(A)** NAC-CD vs. all other groups and NAC-HFD vs. all other groups; *p < 0.05 **(B)** 30 min, NAC-HFD vs. all other groups.

In males, NAC led to overall reduced CORT response to restraint stress (main effect of NAC: *F*_(1,29)_ = 36.729; *p* < 0.0001). This decrease was stronger in NAC-CD subjects, maternal HFD reducing this effect (*F*_(3,87)_ = 6.344, *p* = 0.0006, *post hoc* *p* < 0.001, Figure 5A).

As for females, no main effects of treatment (*F*_(1,26)_ = 0.182; *p* = 0.6728) or diet (*F*_(1,26)_ = 0.096, *p* = 0.7590) were found. In females, the combination of prenatal NAC and HFD led to a reduction in CORT levels comparable to that observed in NAC-HFD males (*F*_(1,26)_ = 8.867, *p* = 0.0062, *post hoc* *p* < 0.05, Figure 5B).

### Glutathione Levels in Hypothalamus and Brown Adipose Tissue (BAT)

**Figure 6:**
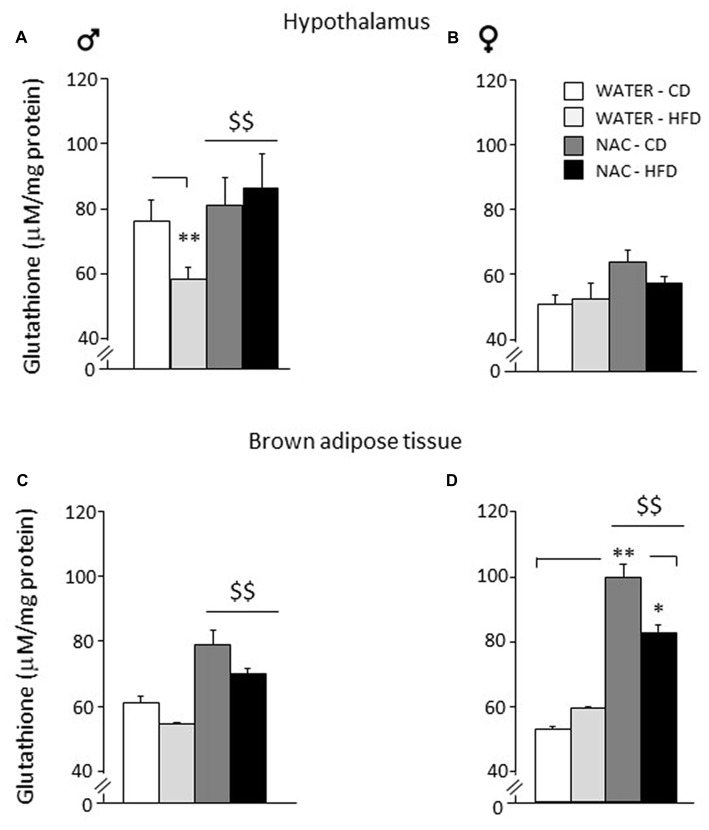
Levels of glutathione in peripheral and central tissues. Hypothalamus: **(A)** glutathione levels were lowered by diet in male subjects (WATER-HFD). Independently from prenatal HFD, all males showed higher glutathione levels as a result of NAC administration. **(B)** Females mice showed reduced glutathione levels in this area and appeared to be more resilient to changes either upon HFD or NAC. Brown Adipose Tissue (BAT): **(C)** Both male and female subjects showed an increase in glutathione levels upon NAC administration. **(D)** In addition, females show a significant increase in glutathione (NAC-CD) that was reduced upon HFD administration (NAC-HFD). Data are mean + SEM. **p < 0.01 for WATER-HFD vs. WATER-CD in male **(A)** and NAC-CD vs. WATER-CD **(D)**; *p < 0.05 for NAC-HFD vs. NAC-CD **(D)**. ^$$^*p* < 0.01 main effect of NAC **(A,C,D)**.

As for the hypothalamus, a main effect of sex showed that females were characterized by reduced levels of glutathione in this brain area (*F*_(1,24)_ = 215.817; *p* < 0.0001). NAC led to an increase in the levels of total glutathione in males but not in females (main effect of NAC: *F*_(1,24)_ = 16.807; *p* = 0.0004). Furthermore, an interaction among sex, treatment and diet was found with male WATER-HFD subjects showing reduced glutathione levels when compared to the control group (*F*_(1,24)_ = 5.526; *p* = 0.0273; *post hoc*: WATER-HFD vs. WATER-CD, *p* < 0.01; see Figures 6A,B).

As for Brown Adipose Tissue (BAT), a main effect of sex showed that females were characterized by increased glutathione levels in this fat depot (*F*_(1,16)_ = 5.485; *p* = 0.0324. NAC led to an increase in the levels of total glutathione both in males and in females (main effect of NAC: *F*_(1,16)_ = 27.310; *p* < 0.0001). In this regard, it is interesting to note that the profile of total glutathione observed in males in this fat compartment, was very similar to that observed in the hypothalamus. In addition, an interaction among sex, treatment and diet was found (*F*_(1,16)_ = 4.460; *p* = 0.0508) showing higher glutathione levels in the NAC-CD females when compared to their controls (*post hoc*: NAC-CD vs. WATER-CD, *p* < 0.01), such effect was reduced in the NAC-HFD group (*post hoc*: NAC-CD vs. NAC-HFD, *p* < 0.05; see Figures 6C,D).

### Emotional Phenotype

#### Open Field (OF)

**Figure 7:**
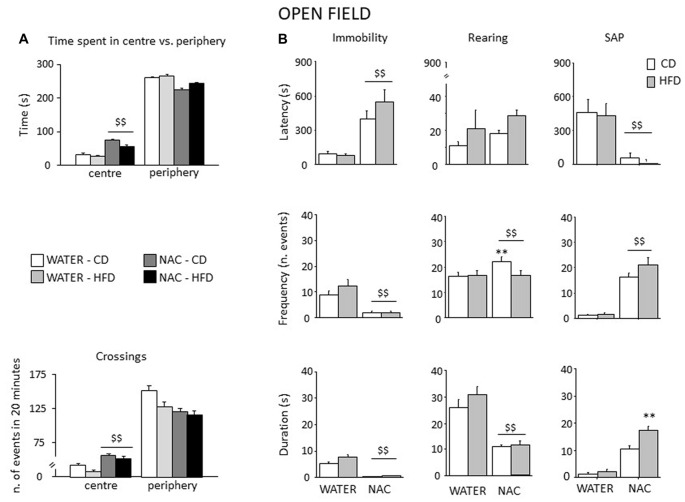
Spontaneous behavior in the OF test. In general, NAC led to a reduced emotionality and increased exploration in the OF test **(A)**. NAC-treated subject spent more time and were characterized by increased locomotion in the central part of the arena when compared to controls. In the plus-maze test, NAC mice showed an increased latency and a reduced frequency and duration of immobility **(B)**. NAC increased rearing bouts, particularly in CD subjects while decreasing the overall duration of this behavior **(B)**. In addition, the latency to the first risk-assessment episode (stretched-attend posture—SAP) was strongly reduced in NAC-treated mice while the frequency was increased; the combination of prenatal NAC and HFD increased the duration of the stretched-attend-posture behavior **(B)**. Data are mean + SEM ^$$^*p* < 0.01, NAC vs. WATER; **p < 0.01; NAC-CD vs. NAC-HFD.

All mice spent more time in the periphery of the arena (main effect of zone: *F*_(1,52)_ = 2293.444, *p* < 0.0001). By contrast, prenatal NAC administration increased the time spent in the center of the OF (interaction between treatment and zone: *F*_(1,52)_ = 59.384, *p* < 0.0001) as well as the locomotor activity (interaction between zone and treatment for crossing frequency: *F*_(1,52)_ = 23.574, *p* < 0.0001); see Figure 7A. Overall, NAC-treated mice appeared to be more explorative. Indeed, they showed an increased latency to first immobility bout as well as a reduced frequency and duration of this behavior (main effect of treatment: *F*_(1,52)_ = 63.096, 46.871, 40.927; *p* < 0.0001 respectively for latency, frequency and duration, Figure 7B). Moreover, all NAC-treated subjects were characterized by increased rearing frequency, particularly NAC-CD mice (interaction between diet and treatment: *F*_(1,52)_ = 5.296; *p* = 0.0254) and by a reduced duration of this behavior (main effect of treatment (*F*_(1,52)_ = 138.022; *p* < 0.0001, Figure 7B). No effects were observed on rearing latency (main effect of treatment: *F*_(1,52)_ = 1.262; *p* = 0.2664). Treatment with NAC affected also SAP behavior. In fact, all mice showed a reduce latency and increased frequency and duration of this behavior (main effect of treatment: *F*_(1,52)_ = 21.134, 132.342, 143.809; *p* < 0.0001, respectively for latency, frequency and duration; see Figure 7B). As for SAP duration, the effect was particularly apparent in the NAC-HFD group (*F*_(1,52)_ = 10.322; *p* = 0.023, *post hoc*: WATER-HFD vs. NAC-HFD *p* < 0.01; Figure 7B). Moreover, prenatal NAC treatment resulted in increased grooming frequency (main effect of treatment: *F*_(1,52)_ = 50.174; *p* < 0.0001) but no effect was observed on latency (*F*_(1,52)_ = 0.621, 0.374 and duration (*F*_(1,52)_ = 0.374; *p* = 0.5436).

#### Elevated Plus Maze (EPM)

**Figure 8:**
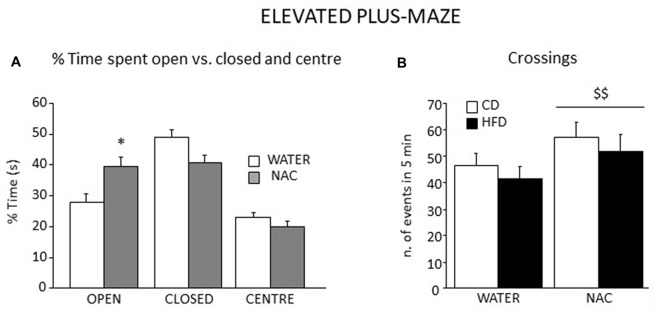
Emotional behavior in the EPM test. All subjects spent more time in the closed arms of the maze although a significant interaction between zone and prenatal treatment revealed that NAC mice spent significantly more time in the open arms when compared to their controls **(A)** In addition, all NAC subjects were characterized by increased locomotion (n. of crossings, **B**). Data are mean + SEM. *p < 0.05, open arms: NAC vs. WATER; ^$$^*p* < 0.01, NAC vs. WATER.

Overall, all subjects spent more time in the closed arms of the apparatus (main effect of zone: *F*_(2, 106)_ = 27.553, *p* < 0.0001). An interaction between zone an treatment showed that, when compared to controls, NAC-treated mice spent more time in the open arms of the maze (*F*_(2,106)_ = 5.499; *p* = 0.0053; Figure 8A). Regardless of both maternal diet and sex, NAC mice showed an increased locomotion when compared to controls (main effect of NAC on frequency of crossing: *F*_(1,53)_ = 5.666, *p* = 0.0209, see Figure 8B), as well an increased frequency of SAP, which was also found in the HFD offspring (main effects of NAC and maternal diet respectively: *F*_(1,53)_ = 5.620; 4.357, *p* = 0.0214; 0.0417).

While NAC, maternal diet and sex did not affect the latency to the first episode of HEAD (main effects of NAC, maternal diet and sex respectively: *F*_(1,53)_ = 2.009; 0.289; 0.129, *p* = 0.1623; 0.5931; 0.7209), an interaction among them was found, with NAC-CD females showing a longer latency than males (*F*_(1,53)_ = 4.428, *p* = 0.0401). Moreover, NAC mice performed HEAD with a reduced frequency than controls (main effect of NAC: *F*_(1,53)_ = 14.404, *p* = 0.0004). The NAC group also showed increased latency to the first episode of grooming behavior not affecting its frequency and duration (main effect of NAC: *F*_(1,53)_ = 4.824; 0.135; 1.345, *p* = 0.0325; 0.7152; 0.2514, respectively for latency, frequency and duration).

## Discussion

Overall, results from this study show that prenatal NAC administration counteracts some of the short- and long-term negative effects observed following *in utero* exposure to HFD. As an example, in females, NAC administration was able to reduce high levels of leptin resulting from prenatal HFD. Prenatal NAC administration also resulted in greater glucose tolerance and insulin sensitivity while increasing adiponectin levels, as well as increasing exploratory behavior, an effect accompanied by a reduced plasma corticosterone levels in response to restraint stress. Analysis of glutathione—an endogenous substance with antioxidant properties—in the hypothalamus and in brown adipose tissue indicates that, while HFD administration to pregnant dams led to lower levels of glutathione in the male hypothalamus, NAC was able to revert this effect both in the periphery (BAT, both males and females) and in the CNS (males). This explains some of the positive long-term and sex-dependent effects of NAC administration *per se*. These data are in line with previous results from our group suggesting that the oxidative *milieu* during pregnancy can have important health outcomes on the offspring (Berry and Cirulli, 2013; Bellisario et al., 2014).

Exposure to HFD feeding during pregnancy represents a metabolic stressful challenge negatively affecting physiological adaptation to pregnancy. In this study maternal HFD led to an overall increase in body weight in all dams that was apparent in the offspring on PND 30, an effect possibly potentiated by weaning as previously shown with an equivalent animal model (Bellisario et al., 2014). NAC administration alone resulted in increased birth weight of the offspring on PND 3 and at adulthood PND 90.

When circulating adipokines were measured, prenatal HFD resulted in increased leptin levels in females, a phenomenon we have previously described (Bellisario et al., 2014). Here we show that this effect can be prevented by maternal NAC administration. In addition, NAC led to an overall increase in adiponectin levels, particularly in males. Leptin and adiponectin are two major adipokines involved in the control of metabolic homeostasis. Leptin affects food consumption and energy expenditure (Friedman and Halaas, 1998) acting directly on the hypothalamus (a brain region involved in energy homeostasis) and hyperleptinemia has been associated to obesity and the related disorders (Considine et al., 1996). By contrast, adiponectin increases tissue fat oxidation, leading to reduced levels of fatty acids and tissue triglyceride content, overall increasing insulin sensitivity (Matsuzawa et al., 2004). Thus, at least as far as adipokines are concerned, we can hypothesize an increased developmental flexibility of females offspring resulting both in an elevated vulnerability to a suboptimal metabolic intrauterine environment as well as in a more efficient response to NAC treatment, while males might be characterized by a higher metabolic resilience.

The interpretation of metabolic assessments at adulthood is more complex. In particular, HFD did not affect, *per se*, glucose tolerance or insulin sensitivity. While in males NAC alone (NAC-CD group) led to a more effective glycemic profile, as assessed both in the GTT and IST, this was not observed in females. In the case of insulin sensitivity, it appears that subjects experiencing prenatal NAC in combination with HFD were characterized by decreased insulin sensitivity. A potential explanation for this result comes from the consideration that, although the excessive production of ROS is associated with many human diseases, in recent years it has become apparent that low levels of H_2_O_2_ may in fact be required for normal cellular functioning and intracellular signaling (Veal et al., 2007; see also further in the “Discussion” section).

Assessment of HPA axis reactivity showed that, while HFD did not affect, *per se*, the neuroendocrine response to a psychophysical stress (restraint), NAC, alone (males) or in combination with HFD (females), overall led to a blunted rise in CORT levels in response to stress. Glucocorticoid hormones (GC) show a very broad variety of actions inducing alert responses through the activation of metabolic and behavioral processes (Oitzl et al., 1997). Interestingly, NAC treated subjects were overall characterized by a decreased emotionality as—differently from HFD-mice—they spent more time in the center of the arena, were less immobile and more explorative in the OF test, in agreement with the reduced activation of the HPA axis. In addition, they showed reduced emotionality in the Elevated Plus-Maze test spending more time in the open (anxiogenic) arms of the maze. Previous authors have found a link between genes involved in the regulation of OS and emotionality suggesting a tight relationship between OS metabolic pathways and normal anxiety (Hovatta et al., 2005). When compared with previous data obtained in other animal model, such as the p66Shc^−/−^ mouse (Berry et al., 2007, 2008; Berry and Cirulli, 2013) these results strengthen the notion of a link among OS, metabolism and emotional behavior, indicating a potential role for antioxidants in the management of anxiety and/or psychiatric disorders (Giorgio et al., 2012; Berry and Cirulli, 2013).

Overall, in this study, while prenatal HFD did not show pervasive effects on the metabolic or emotional profile of the experimental subjects, NAC-treated mice showed improved glucose tolerance in addition to a reduced activation of the HPA axis upon restraint stress. Moreover, when tested in the OF and in the Elevated Plus-Maze, they were characterized by a reduced emotional profile. Intriguingly, the observed effects of prenatal NAC mirror the overall phenotype of p66Shc knock-out mice previously described (Berry and Cirulli, 2013; Bellisario et al., 2014). Since this is an animal model of reduced OS and healthy aging, these data might suggest that the prenatal-NAC-phenotypic features might be protective upon further metabolic or stressful challenges that could be experienced at adult age. This hypothesis is strengthened by data showing meaningful effects of NAC on glutathione levels, a powerful antioxidant and a ROS scavenger, in central (hypothalamus) and peripheral (brown fat) tissues. Data indicate that, while HFD administration to pregnant dams leads to reduced levels of glutathione in male hypothalamus, NAC is able to revert this effect and to increase levels of glutathione in the periphery (BAT, both males and females). Females appear overall more resistant to changes in glutathione levels in the CNS compared to males, while the opposite holds true in the periphery.

Tight regulation of the redox balance, particularly in a developing organism is crucial for the maintenance of proper cell function (Ristow et al., 2009). Although antioxidant therapy is currently emerging as a promising strategy to counteract the prevalence of diabetes and other metabolism-related pathologies, in addition to anxiety and psychiatric disorders, unfortunately, major randomized clinical trials have yielded conflicting results (Falach-Malik et al., 2016). Results from this study, performed in an animal model, indicate positive effects of NAC given during pregnancy on the developing offspring although they also raise some questions concerning safety. A wide variety of stimuli including growth factors and hormones can promote the transient generation of H_2_O_2_, and recent data provide clear evidence for the enhancement of insulin signaling by ROS *in vivo* (Loh et al., 2009). This might suggest that lowering excessively ROS might endanger signal transduction mechanisms they regulate.

Overall, these data indicate that a major challenge for future investigations will be to determine the optimal dose of compounds capable to keep the redox balance within an optimal range, taking into account physiological functions involving redox-sensitive pathways, age- and sex/gender differences.

## Author Contributions

AB and FC designed the experiments, supervised all the experimental procedures and drafted the article. VB and PP performed all the behavioral and neuroendocrine assessments. CR performed all the biochemical assays, carried-out the statistical analysis and drafted the article. MA and MCM performed glutathione biochemical assay and carried-out statistical analysis and data interpretation on this assay.

## Conflict of Interest Statement

The authors declare that the research was conducted in the absence of any commercial or financial relationships that could be construed as a potential conflict of interest.
